# Supplementary material for: Core@Shell AgBr@CsPbBr3 Nanocrystals as Precursors to Hollow Lead Halide Perovskite Nanocubes
Source: J Am Chem Soc. 2025 Jun 16;147(26):23192–201. doi: 10.1021/jacs.5c07200 (PMC12232317; doi:10.1021/jacs.5c07200)
Supplement: Supplementary file 1 [file ja5c07200_si_001.pdf]

Supporting Information for:

Core@Shell $AgBr@CsPbBr_3$ Nanocrystals as Precursors to Hollow Lead Halide Perovskite Nanocubes

Zhanzhao Li[1], Yurii P. Ivanov[2], Anna Cabona[1,3], Andrea Fratelli[1,4], Stefano Toso,[1] Saptarshi Chakraborty[4], Giorgio Divitini[2*], Ilka Kriegel[3], Sergio Brovelli[4*], Liberato Manna[1*]

[1] Nanochemistry, Istituto Italiano di Tecnologia, Via Morego 30, 16163, Genova, Italy

[2] Electron Spectroscopy and Nanoscopy, Istituto Italiano di Tecnologia, Via Morego 30, 16163, Genova, Italy

[3] Department of Applied Science and Technology, Politecnico di Torino, Corso Duca degli Abruzzi 34, 10129, Turin, Italy

[4] Dipartimento di Scienza dei Materiali, Università degli Studi di Milano Bicocca, via R. Cozzi 55, 20125, Milano, Italy

Contents: Figures S1-S16, Tables S1-S3, additional references

Epitaxial structure matching between AgBr and $CsPbBr_3$.

The likelihood of a successful growth of AgBr@$CsPbBr_3$ core-shell nanoparticles was evaluated by simulating the possible AgBr/$CsPbBr_3$ epitaxial relations, using the Ogre library for the prediction of interfaces between ionic materials. Due to the small deviations of the orthorhombic Pnma $CsPbBr_3$ structure from the Pm-3m cubic perovskite prototype, in our simulations, we initially considered using the latter case in $CsPbBr_3$ structure simulation, which allows for a more intuitive interpretation of results.

The key requirements for growing an epitaxial shell are:

- 1) That the two materials can form stable interfaces along multiple directions of their lattice, to ensure that the seed material can be fully enveloped.
- 2) That these interfaces form by retaining the same relative orientations of the two atomic lattices, to ensure that the outer shell can grow as a single-crystalline domain.

The lattice matching table in **Figure S1** (see Ref.[1] for details on the interpretation) clearly indicates that these conditions can be met for AgBr/$CsPbBr_3$, as both materials are cubic and all interfaces where $(hkl)_{AgBr} = (hkl)_{CsPbBr3}$ display favorable geometric matching with small interface supercells and low strain of 1.16%.

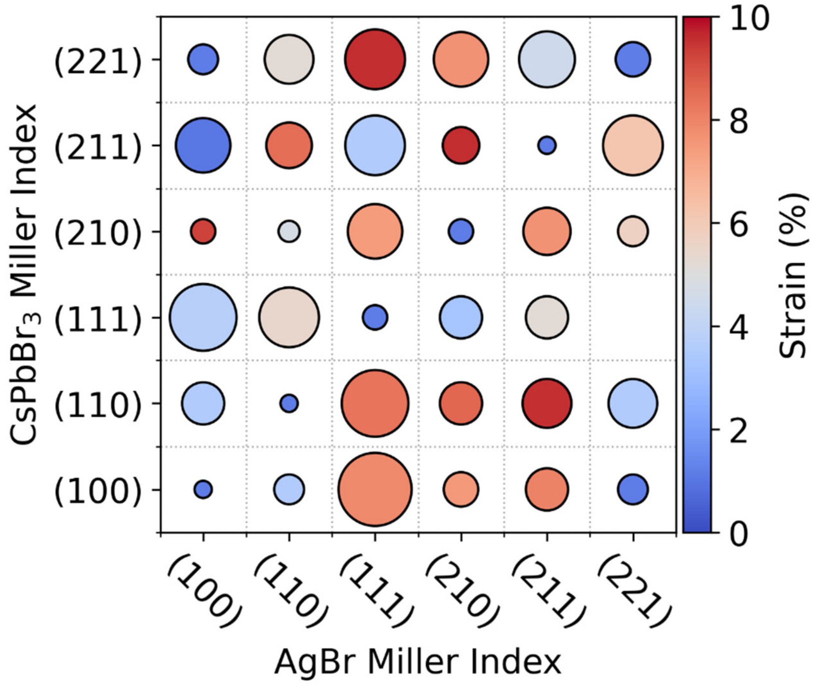

Figure S1. Lattice matching table for the AgBr/cubic-$CsPbBr_3$ pair. Each circle summarizes the geometric properties of the corresponding (hkl)/(h'k'l) interface. The circle area indicates the interface supercell surface (smaller = better), while the circle color indicates the interface strain (blue = better).

Simulations of the possible interface structures, performed by considering (hkl) = (h'k'l) up to h,k,l = 2, indicate that all crystallographic directions can give rise to stable interfaces between AgBr and $CsPbBr_3$ (**Table S1**), attesting to their excellent structural compatibility.

Table S1. Predicted geometric parameters and interface energies for AgBr/$CsPbBr_3$ interfaces of the (hkl) = (h'k'l) type.

(hkl) AgBr	(hkl) $CsPbBr_3$	Strain (%)	Area /Å²	Interface Energy meV/Å²
(100)	(100)	1.16	33.7	5.1
(110)	(110)	1.16	47.7	42.0
(111)	(111)	1.16	58.4	47.6
(210)	(210)	1.16	150.7	22.1
(211)	(211)	1.16	82.6	49.8
(221)	(221)	1.16	199.9	17.7

However, the interface energy for the (100)/(100) AgBr/$CsPbBr_3$ interface (**Figure S2**) is significantly lower than that of any other direction. Interpreting this result in the light of the Wulff construction for crystals with minimal surface energy leads to the conclusion that the most likely shape for an AgBr seed embedded inside a $CsPbBr_3$ domain is that of a sharp cube exposing preeminently its (100) facets.

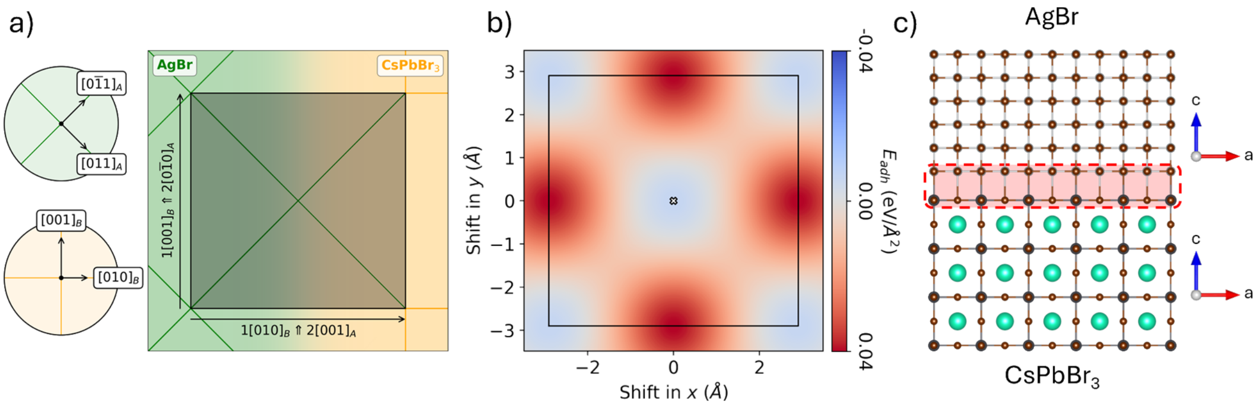

Figure S2. (100)/(100) AgBr/$CsPbBr_3$ interface. a) Supercell scheme, showing the relative orientation of the AgBr and $CsPbBr_3$ lattice vectors at the interface. b) Potential energy surface describing the relative in-plane shift of the two materials which leads to the most stable interface configuration. c) Atomic model of the interface as produced by Ogre, showing that the most favorable termination for $CsPbBr_3$ at the interface is the $PbBr_2$ plane, which leads to the formation of Pb-Br and Ag-Br bonds at the interface (highlighted in red).

Control simulations performed considering the orthorhombic Pnma structure for $CsPbBr_3$ instead of the simplified Pm-3m confirmed the same results, with minimal stability differences between the (100)/(010) AgBr/Pnma-$CsPbBr_3$ interface (9.5 meV/Å²) and the (100)/(101) AgBr/Pnma-$CsPbBr_3$ interface (9.3 meV/Å²). In the interest of structural accuracy, an interface model displaying the orthorhombic structure for $CsPbBr_3$ is shown as a part of **Scheme 1b** in the Main text.

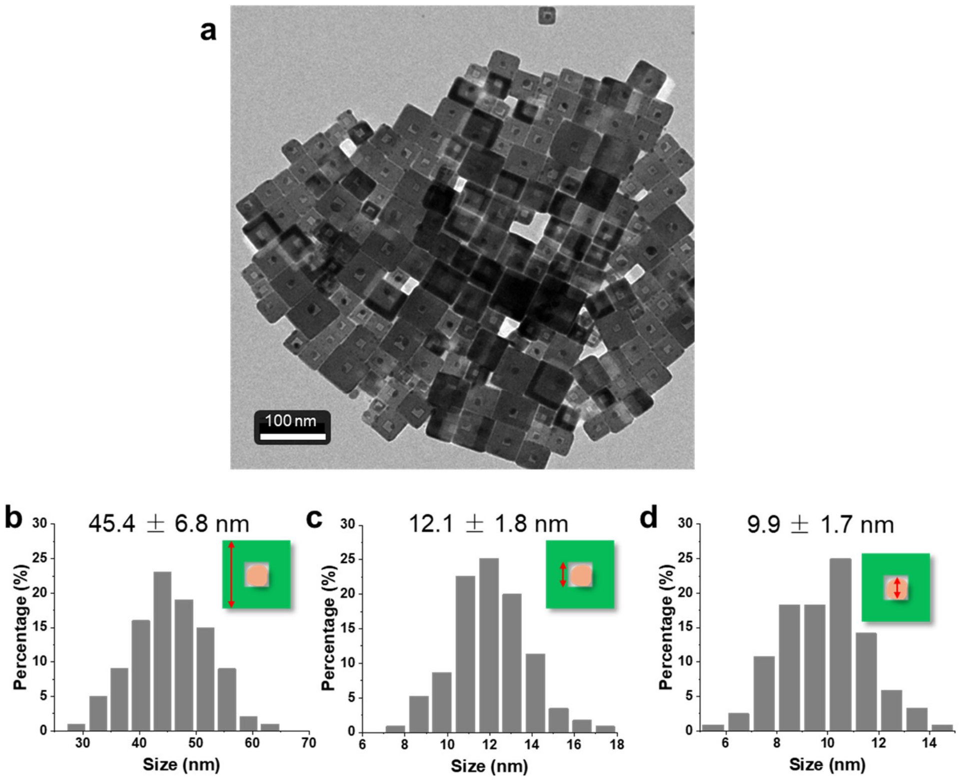

Figure S3. (a) TEM image of NCs synthesized with Zn^{2+} addition (molar ratios of Cs:Pb:Ag:Zn = 1:1:2:0.25). (b, c, d) Size distributions of the nanocube, cavity, and solid core region.

Table S2. Ag:Pb atomic ratio in $AgBr@CsPbBr_3$, $Ag@CsPbCl_3$ and hollow $CsPbI_3$ NCs according to ICP-OES analysis.

	Element content (ppm)		
Samples	Pb	Ag	Ag:Pb
$AgBr@CsPbBr_3$	1.09	0.11	0.10
$Ag@CsPbCl_3$	1.69	0.11	0.07
Hollow $CsPbI_3$	1.4	0	-

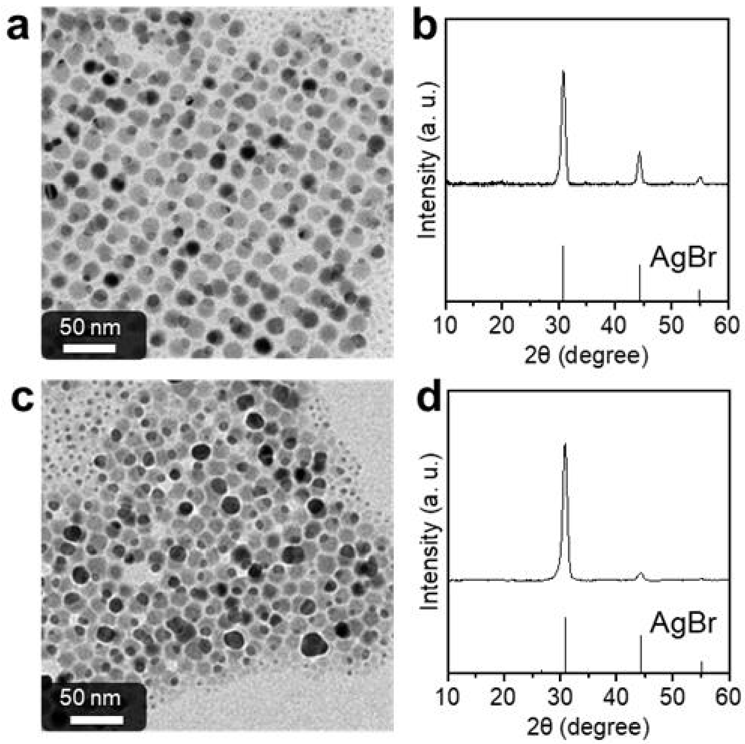

Figure S4. (a, c) TEM images, and (b, d) XRD patterns of NCs synthesized (a, b) without Zn^{2+} addition and (c, d) with Zn^{2+} addition (Cs:Pb:Ag:Zn=1:1:2:0.25). In both cases, the synthesis was stopped 10 sec after the injection. In (b) and (c), the tabulated reflections for AgBr (ICSD number 56546) are marked by vertical black bars. In both cases, AgBr NCs were synthesized.

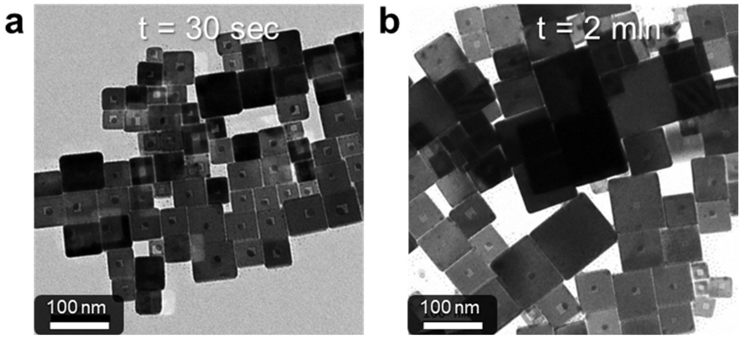

Figure S5. TEM images of samples recovered at (a) 30 sec and (b) 2 min after the injection (molar ratios of Cs:Pb:Ag:Zn = 1:1:2:0.25).

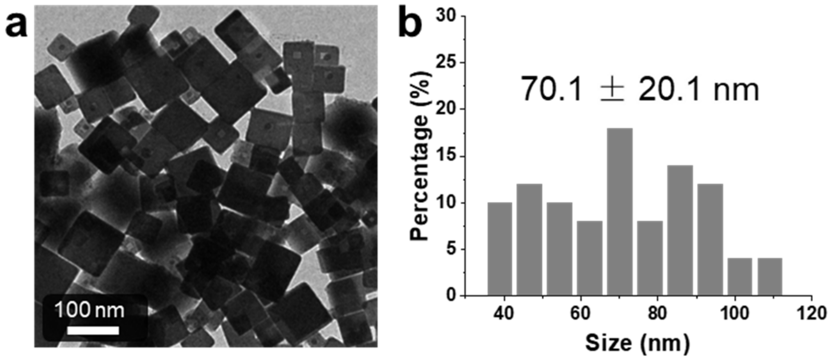

Figure S6. (a) TEM images and (b) size distribution of NCs synthesized without Zn^{2+} addition (molar ratios of Cs:Pb:Ag = 1:1:2).

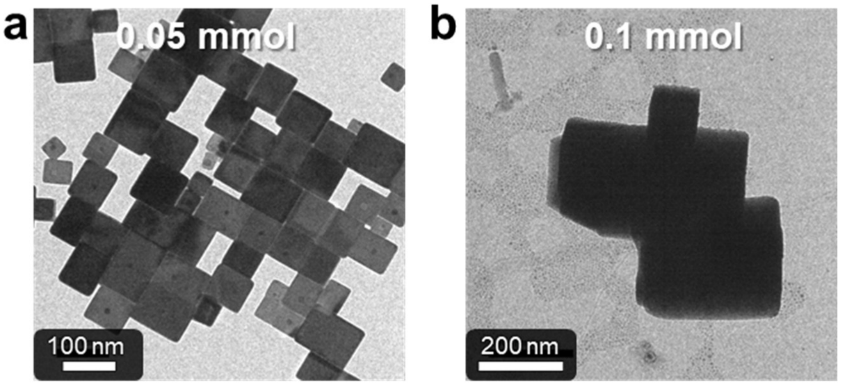

Figure S7. TEM images of samples obtained with (a) 0.05 mmol and (b) 0.1 mmol Zn^{2+} addition (molar ratios of Cs:Pb:Ag = 1:1:2).

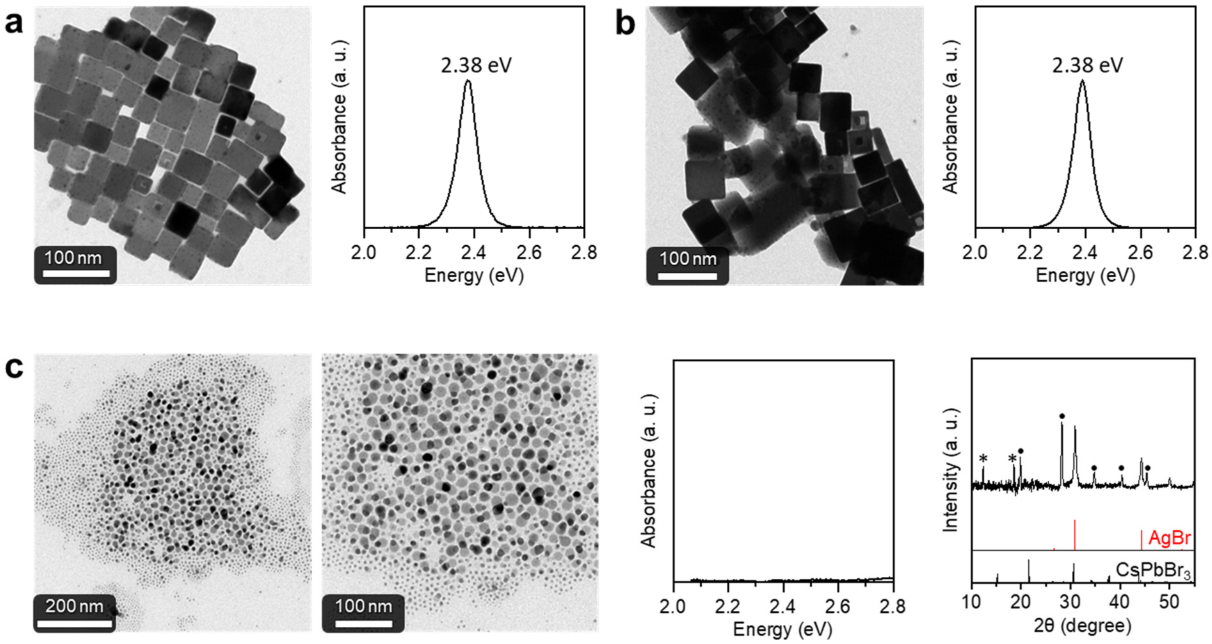

Figure S8. TEM, PL spectra and XRD patterns of samples obtained with (a) 0.15 mmol, (b) 0.25 mmol and (c) 0.3 mmol Ag^+ addition (molar ratios of Cs:Pb:Zn = 1:1:0.25). In the XRD pattern analysis in panel c, except for the peaks assigned to AgBr, additional peaks are assigned to $PbBr_2$ (marked by *) and $AgNO_3$ (marked by •).

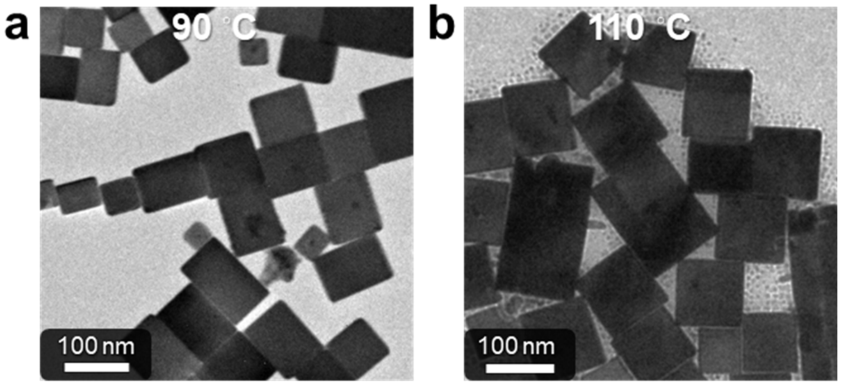

Figure S9. TEM images of samples synthesized at reaction temperatures of (a) 90°C and (b) 110°C (molar ratios of Cs:Pb:Ag:Zn = 1:1:2:0.25).

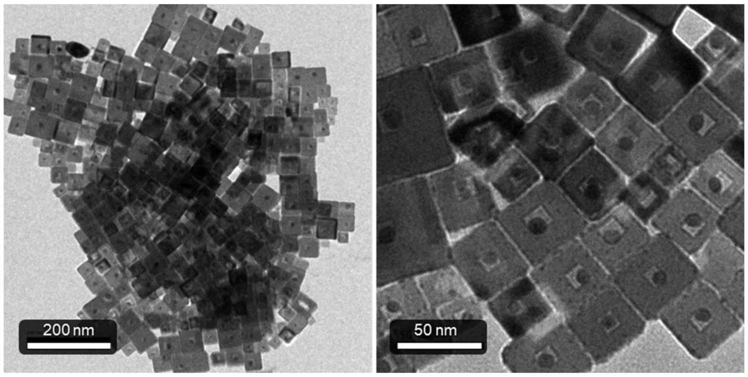

Figure S10. TEM images of $Ag@CsPbCl_3$ NCs prepared by Cl^- exchange on the $AgBr@CsPbBr_3$ NCs.

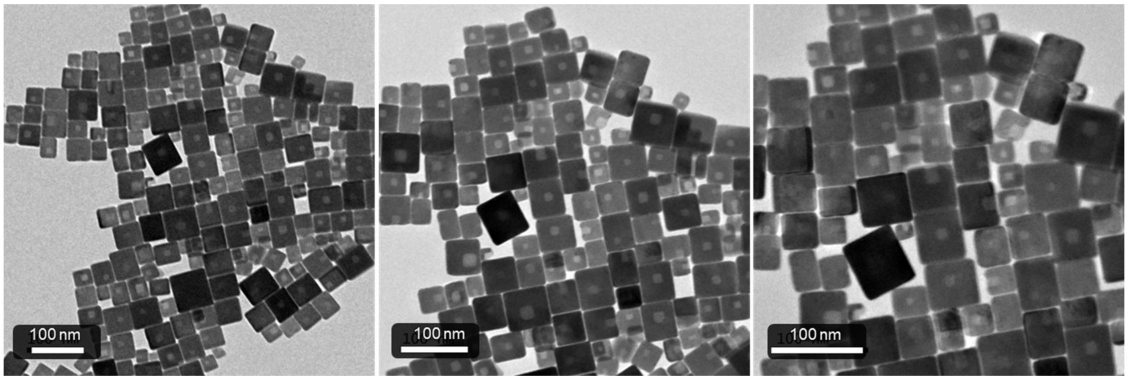

Figure S11. TEM images of hollow $CsPbI_3$ NCs prepared by I^- exchange on the $AgBr@CsPbBr_3$ NCs.

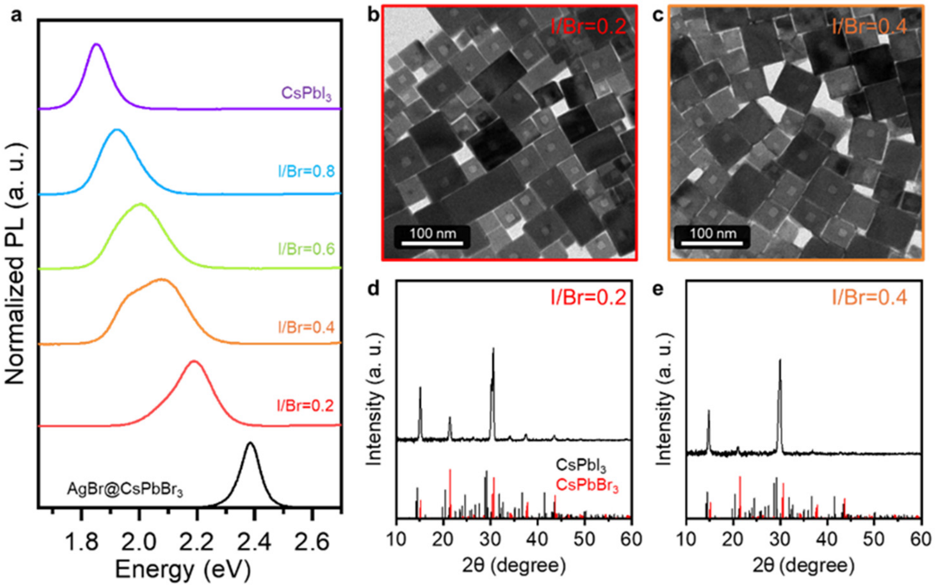

Figure S12. Br^- to I^- exchange on core@shell $Ag@CsPbBr_3$ NCs. (a) PL spectra of the starting, intermediate and final samples for the Br^- to I^- exchange reaction. (b, c) TEM images and (d, e) XRD patterns of a couple of intermediate exchange samples: (b, d) I/Br = 0.2 and (c, e) I/Br = 0.4, which resulted from sequential aliquots of iodide being added in the same reaction. In (d, e), the reflections of $CsPbBr_3$ (ICSD number 97851) and $CsPbI_3$ (ICSD number 69423) are represented by vertical black and red bars, respectively.

Table S3. ICP-OES analysis of the supernatant solution after the I^- exchange reaction on the AgBr@$CsPbBr_3$ NCs. In the I^- exchange reaction, 0.5 mL of a colloidal suspension of AgBr@$CsPbBr_3$ NCs (5 mM in Pb) was added to 2 mL of toluene, then 0.5 mL of iodine precursor (25 mM Pb) solution was added. The NCs were then separated and the supernatant was analyzed. The experimental Ag:Pb ratio was 0.018 (0.02/1.09), which is very close to the estimated Ag:Pb ratio of 0.02 in case all the Ag initially present in the AgBr@$CsPbBr_3$ NC was released into the solution and thus in the supernatant.

Element	Content (ppm)
Pb	1.09
Ag	0.02

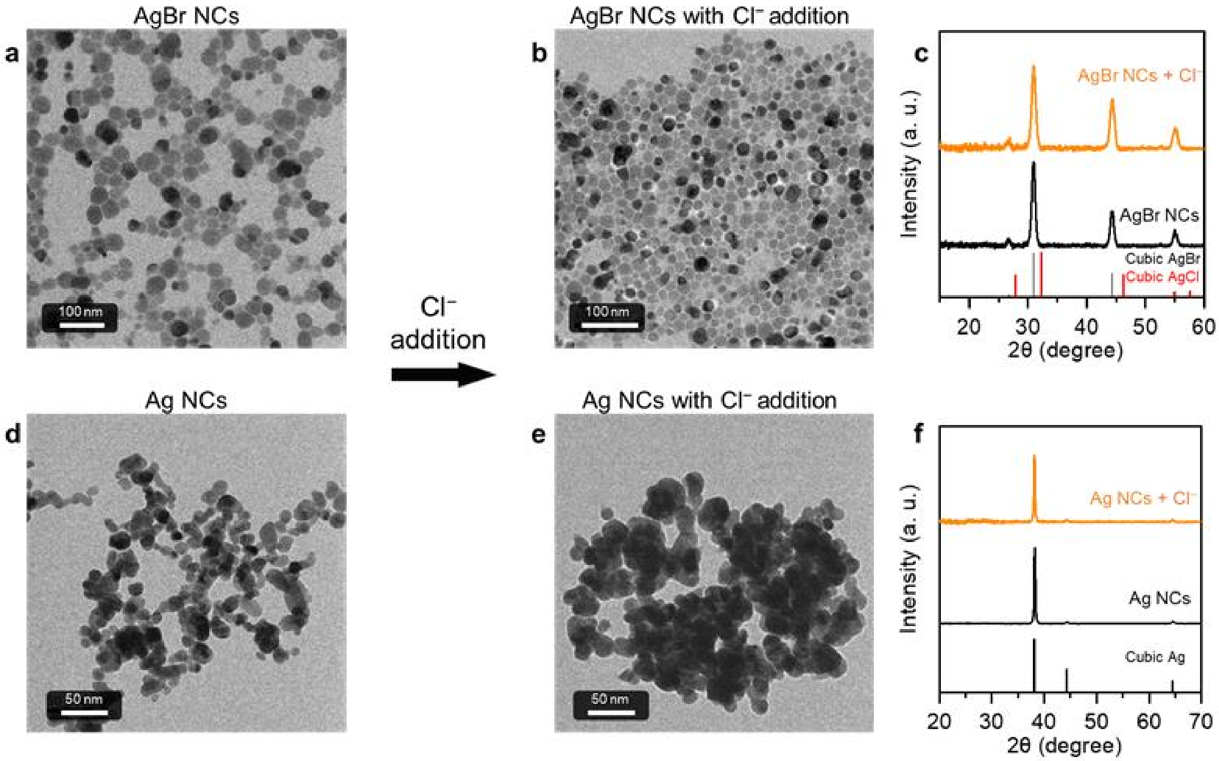

Figure S13. Results of Cl^- addition to a colloidal suspension of AgBr NCs (top panels) and Ag NCs (bottom panels). (a, b) TEM images and (c) XRD pattens of the (a) starting AgBr NCs and (b) resulting NCs after Cl^- addition. (d, e) TEM images and (f) XRD pattens of the (e) starting Ag NCs and (f) resulting NCs after Cl^- addition. In (c), the reflections of cubic AgCl (ICSD number 56548) are represented by vertical red bars. In (f), the reflections of Ag (ICSD number 13762) are represented by vertical black bars.

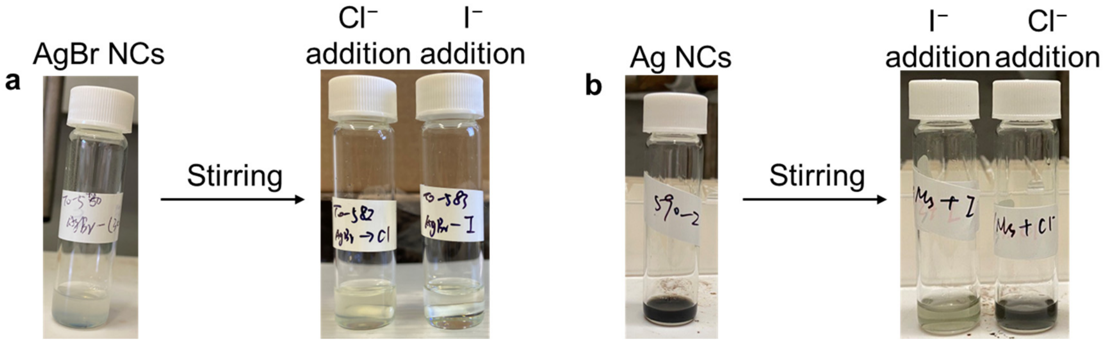

Figure S14. (a, b) Photographs of vials before and after adding I⁻ and Cl⁻ to colloidal suspension of AgBr NCs (a) and Ag NCs (b). In the Cl⁻ cases, we identified the AgBr or Ag NCs in the solutions, as discussed in Figure S13. In the I⁻ cases, both suspensions became transparent after I⁻ addition, and no precipitate was recovered after centrifugation. No NCs could be identified by TEM observations of these two latter solutions.

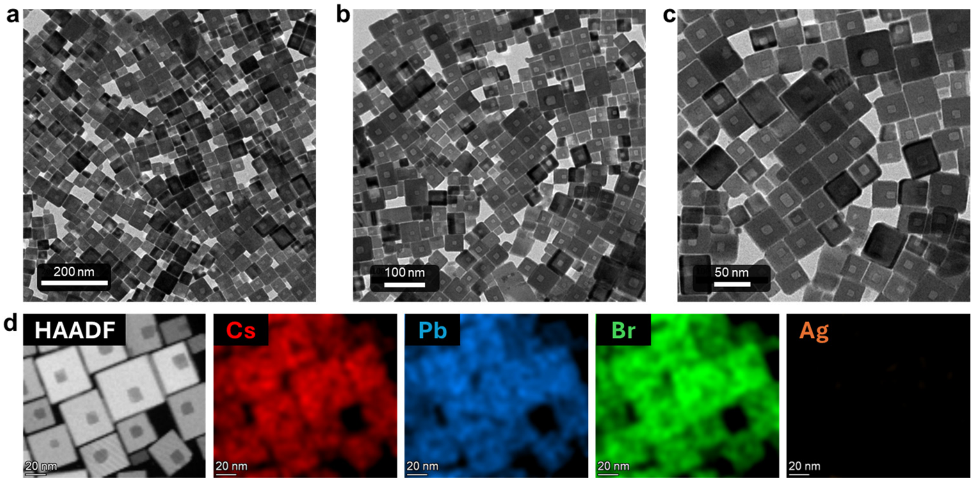

Figure S15. (a-c) TEM images and (d) STEM-EDX maps of hollow $CsPbBr_3$ NCs.

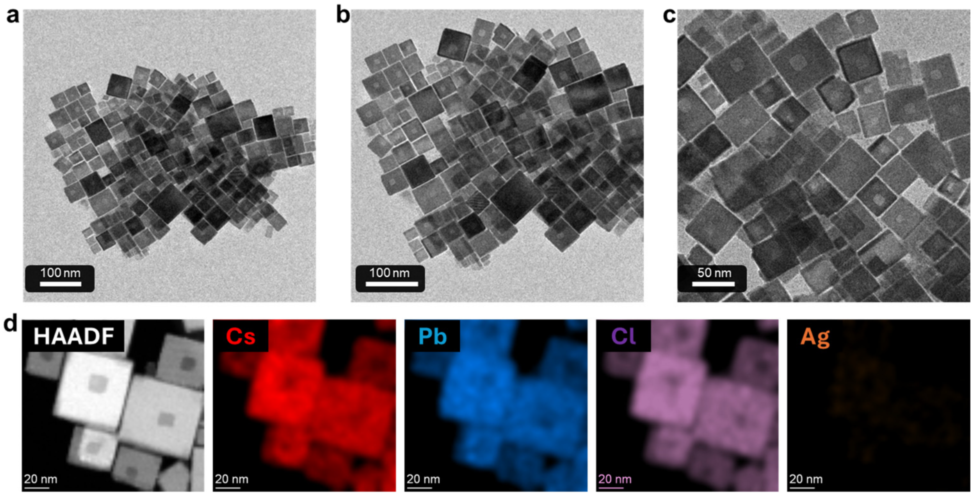

Figure S16. (a-c) TEM images and (d) STEM-EDX maps of hollow $CsPbCl_3$ NCs.

ADDITIONAL REFERENCES

(1) Toso, S.; Dardzinski, D.; Manna, L.; Marom, N. Structure Prediction of Ionic Epitaxial Interfaces with Ogre Demonstrated for Colloidal Heterostructures of Lead Halide Perovskites. ACS Nano **2025**, *19* (15), 5326–5341.
